# Comprehensive ultrasound assessment of thumb metacarpophalangeal collateral ligament injuries reveals frequent concomitant abnormalities

**DOI:** 10.1007/s00402-026-06430-0

**Published:** 2026-07-28

**Authors:** Orit Bain, Uri Farkash, Ran Atzmon, Shai Shemesh, Nissim Ohana

**Affiliations:** 1 Department of Orthopaedic Surgery, Meir Medical Center, Kfar Saba, Israel; 2 Maccabi Health Care Services, Tel Aviv, Israel; 3 Department of Orthopaedic Surgery, Assuta Ashdod Medical Center, Ashdod, Israel; 4 Ben-Gurion University of the Negev, Beersheba, Israel; 5 Gray School of Medicine, Tel Aviv University, Tel Aviv, Israel

**Keywords:** Skier’s thumb, Stener lesion, Radial collateral ligament, Extensor mechanism

## Abstract

**Introduction:**

Ulnar collateral ligament (UCL) tears of the thumb are well-recognized injuries, while radial collateral ligament (RCL) and dorsal extensor-related abnormalities are less frequently studied. The pattern of concomitant ultrasound abnormalities in patients after thumb trauma is not well documented. The aim of this study was to characterize concomitant UCL, RCL, and dorsal extensor-related abnormalities within a selected cohort referred to thumb ultrasound after trauma, and to explore associations with injury mechanism.

**Materials and methods:**

A retrospective review was performed of 42 consecutive adult patients who underwent high-resolution ultrasound of the thumb metacarpophalangeal joint between April 2014 and February 2025 for suspected ligamentous injury after acute trauma. Patients were included if ultrasound demonstrated pathologic findings in at least one stabilizing structure, UCL or RCL, and the extensor mechanism was systematically assessed. Collateral ligament injuries were graded using standard sonographic criteria, while dorsal extensor-related findings were recorded descriptively. Associations between injury severity and mechanism of injury were tested using chi-square or Fisher’s exact analysis, as appropriate.

**Results:**

UCL pathology was present in 35/42 (83.3%) patients, RCL pathology in 23/42 (54.8%), and dorsal extensor-related abnormalities in 17/42 (40.5%). The severity distribution of UCL injuries was grade I in 15 patients, grade II in 8, grade III in 9, and grade IV in 3; RCL injuries were grade I in 18 patients, grade II in 4, and grade III in 1. Nearly half of patients with UCL injury also demonstrated RCL involvement, and dorsal extensor-related abnormalities accompanied 75% of combined UCL and RCL injuries. No significant associations were observed between trauma mechanism and injury presence or severity (all *p* > 0.22).

**Conclusions:**

In this selected cohort, combined ultrasound abnormalities involving the thumb MCP collateral ligaments and dorsal extensor structures were frequently observed. In suspected thumb MCP injury, assessment of adjacent stabilizing structures may improve diagnostic completeness and patient counseling and follow-up.

## Introduction

Collateral ligament injuries of the thumb metacarpophalangeal (MCP) joint are common, particularly ulnar collateral ligament (UCL) tears, presumably following valgus stress such as skiing accidents or falls onto an outstretched hand [1–4]. Skier’s thumb refers to an acute valgus injury of the thumb UCL, whereas Gamekeeper’s thumb classically describes chronic attenuation of the same ligament. A Stener lesion is a specific acute UCL injury in which the torn ligament becomes displaced superficial to the adductor aponeurosis, preventing spontaneous healing and typically requiring operative treatment. In contrast, injury to the radial collateral ligament (RCL) has only recently gained broader acceptance as an important contributor to stability and function of the metacarpophalangeal (MCP) joint [5, 6].

Accurate diagnosis of MCP injuries relies on imaging; radiographs identify associated fractures, and magnetic resonance imaging (MRI) or ultrasound (US) directly depict ligament integrity [2, 7]. High-resolution US is increasingly used due to its accuracy, availability [8], and ability to evaluate and compare to the contralateral side.

Most prior literature has focused on isolated UCL injuries and their management, while RCL lesions and especially combined UCL–RCL tears are less frequently studied [4, 9]. In elite athletes, combined injuries account for up to 25% of thumb collateral ligament injuries, with involvement of the extensor apparatus described sporadically and not systematically quantified [10]. 

The objective of this study was to assess the frequency and combinations of UCL, RCL, and extensor mechanism abnormalities identified by US in a selected cohort of patients with thumb trauma. We hypothesized that concomitant abnormalities of more than one stabilizing structure would be identified in a substantial proportion of this selected cohort, even when the referral focused on a single suspected ligament.

## Materials and methods

This retrospective study included adult patients who were referred for ultrasound of the thumb MCP joint following acute trauma between April 2014 and February 2025. Inclusion criteria were acute thumb trauma, age 18 years or older, a pathological finding in at least one stabilizing structure, UCL or RCL, and an ultrasound examination performed within 3 months of injury. Exclusion criteria were age under 18 years, open or repetitive injuries, prior thumb surgery or trauma, and rheumatic disease. A total of 54 patients were initially identified during the study period. After application of the predefined inclusion and exclusion criteria, 42 consecutive patients comprised the final analytic cohort. Patients were excluded if they were younger than 18 years or if the medical record contained insufficient data to confirm eligibility or extract the required study variables. The Institutional Review Board approved the study, and informed consent was waived. Demographic data, mechanism of injury, time from trauma to ultrasound, radiographic findings, and sonographic findings were collected from the patient records. Data on hand dominance, standardized clinical stability testing, and prospectively recorded treatment impact were not available in a sufficiently uniform manner for retrospective analysis. Accordingly, the study was designed to describe patterns of concomitant abnormalities within a selected referred cohort rather than to estimate prevalence among all patients presenting after thumb trauma.

Ultrasound examinations were performed by a single orthopedic surgeon with more than 20 years’ experience in diagnostic musculoskeletal US, using a High frequency 6–15 MHz transducer (Logiq 9, GE Medical Imaging, Milwaukee, WI, USA). Since the ultrasound examination was conducted as part of routine clinical care, the examiner was informed of the symptomatic side and the reason for referral. All structures surrounding the MCP joint were evaluated in longitudinal and transverse planes. The extensor apparatus was systematically evaluated [11]. 

The UCL integrity was classified as Grade 0 (normal): homogeneous and fibrillar structure spanning the ulnar side of the first MCPJ; Grade I (sprain): thickening, and hypoechogenicity of the UCL with intact fibers; Grade II (partial-thickness tear): focal hypoechoic/anechoic abnormality that does not involve the entire ligament; Grade III (complete non-displaced tear): focal abnormality involving the entire ligament; Grade IV (Stener lesion): lack of visualization of the UCL with retraction of the ligament proximal to the MCPJ [12]. 

RCL injuries were classified in the same manner as grades 0-III, given that a Stener lesion represents a complication unique to UCL tears [13]. Extensor findings were not graded according to a validated classification system. Instead, they were recorded descriptively as normal, tendon abnormality, or dorsal peritendinous fluid. Tendon abnormality was defined as focal thickening and/or hypoechogenicity of the extensor tendon with preserved continuity. Dorsal peritendinous fluid was defined as fluid adjacent to the extensor tendon on the dorsal aspect of the joint [14]. Dynamic tendon gliding was used during routine examination when needed to help separate the extensor tendon from adjacent dorsal soft tissues. Nevertheless, given the close relationship between the extensor tendon and dorsal capsule at the thumb MCP joint, retrospective distinction between tendon-related and dorsal capsular-related abnormalities was not always possible.

### Statistics

Data analysis was performed using descriptive and inferential statistical methods. Categorical variables are presented as frequencies and percentages. Exact binomial 95% confidence intervals were calculated for the primary frequency estimates. Associations between categorical variables were analyzed using chi-square tests or Fisher’s exact tests when expected cell counts were less than 5. This primarily applied to analyses involving the traction subgroup (*n* = 3) and high-grade RCL abnormalities (*n* = 5), in which expected cell counts were small. To evaluate whether specific mechanisms of injury were associated with higher injury severity or concomitant injuries (i.e., UCL + RCL, UCL+dorsal extensor-related abnormality, RCL+dorsal extensor-related abnormality), subgroup analyses were performed by mechanism category: fall, sports, motor vehicle accident, and traction. A two-sided p-value less than 0.05 was considered statistically significant. All analyses were conducted using IBM SPSS Statistics (IBM Corp., Armonk, NY), version 30.0.

## Results

A total of 54 patients were initially identified during the study period. After application of the predefined inclusion and exclusion criteria, 42 patients comprised the final analytic cohort. The cohort included 23 men and 19 women, with a median age of 40 years (range 19–76). Mechanisms of injury were sports in 17 cases (40.5%), falls in 14 (33.3%), motor vehicle accidents in 8 (19.0%), and traction in 3 (7.1%). All patients had an X-ray of their thumb taken prior to the US exam. In two cases, the radiographs showed a small avulsion fracture of the UCL from the metacarpal head.

**Fig. 1:**
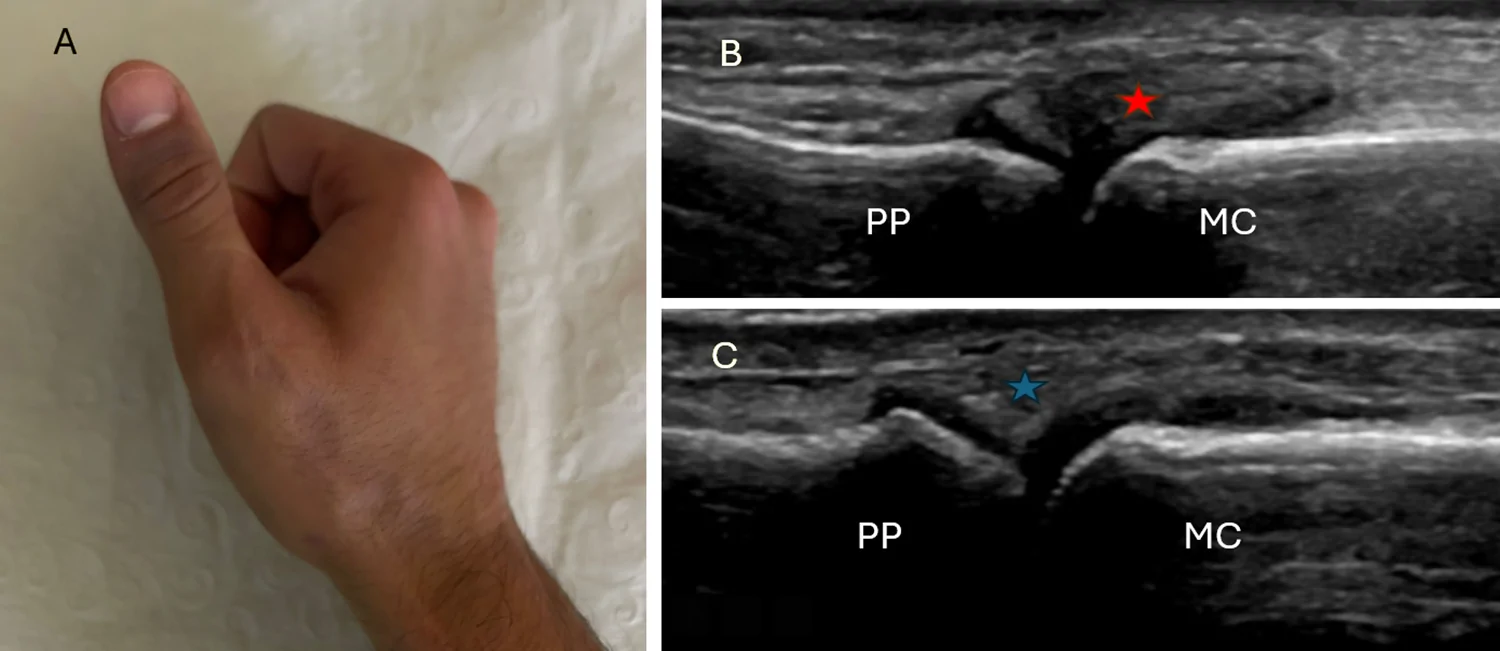
**A**–**C** Thumb collateral ligament injuries in a 27-year-old man after right thumb trauma sustained during soccer. **A** Clinical photograph shows hematoma over the ulnar and dorsal aspects of the MCP joint. **B** Ultrasound image demonstrates a full-thickness UCL tear located beneath the adductor aponeurosis (red asterisk). **C** Ultrasound image demonstrates a thickened but continuous extensor tendon, consistent with dorsal extensor-related abnormality (blue asterisk). *MC* thumb metacarpal; *PP* proximal phalanx

In 35 out of 42 cases (83.3%), an injury of the UCL was identified by US. The severity distribution of UCL injuries was grade I in 15 patients, grade II in 8 patients, grade III in 9 patients, and grade IV in 3 patients. RCL injuries were observed in 23 of 42 cases (54.8%), with 18 classified as grade I, 4 as grade II, and 1 as grade III. Additionally, dorsal extensor-related abnormalities were noted in 17 patients (40.5%), including 15 cases of tendon thickening/hypoechogenicity with preserved continuity and 2 cases of dorsal peritendinous fluid (Table 1). Nearly half of patients with UCL injury (16/35) also demonstrated RCL involvement, and dorsal extensor-related abnormalities were present in 75% (12/16) of combined UCL + RCL cases. Figure 1 demonstrates swelling and ecchymosis along the ulnar and dorsal aspects of the thumb MCP joint in a 27-year-old man following a right thumb injury, raising suspicion for a UCL injury. Ultrasonography demonstrated a complete UCL tear with an associated sprain of the RCL and dorsal extensor-related abnormality (Fig. 1).

**Table 1:** Distribution of ultrasound abnormalities at the thumb MCP joint (*N* = 42)

Finding	UCL, *n* (%)	RCL, *n* (%)	Dorsal extensor-related findings, *n* (%)
Normal	7 (16.7%)	19 (45.2%)	25 (59.5%)
Grade I	15 (35.7%)	18 (42.9%)	–
Grade II	8 (19.0%)	4 (9.5%)	–
Grade III	9 (21.4%)	1 (2.4%)	–
Grade IV (Stener lesion	3 (7.1%)	0 (0.0%)	–
Tendon thickening / hypoechogenicity with preserved continuity	–	–	15 (35.7%)
Dorsal peritendinous fluid	–	–	2 (4.8%)
Total abnormal	35 (83.3%)	23 (54.8%)	17 (40.5%)

*Dorsal extensor-related findings were recorded descriptively rather than according to a validated grading system

Analyzing the distribution of injuries according to mechanism of trauma showed that UCL involvement was frequently observed across all categories, being observed in 78.6% of patients following falls, 76.5% of those injured during sports activities, and in all patients (100%) sustaining motor vehicle accidents or traction injury. RCL injuries were somewhat less consistent, detected in 57.1% of fall-related injuries, 64.7% of sports-related injuries, 50.0% of motor vehicle accident cases, and absent in patients who sustained traction injury. Dorsal extensor-related abnormalities were present in 28.6% of fall-related cases, 41.2% of sports-related cases, 50.0% of motor vehicle accident cases, and in 66.7% of patients with traction mechanism (Table 2). Chi-square analyses found no significant associations between mechanism and injury presence or severity (all *p* > 0.22). A collapsed analysis dichotomizing severity into low-grade (0–1) and high-grade (≥ 2) similarly demonstrated no significant association between injury mechanism and severity for the UCL (*p* = 0.49) or RCL (*p* = 0.92) (Table 3).

**Table 2:** Frequency of ultrasound abnormalities according to mechanism of trauma

Mechanism of injury	Total patients, *N* (%)	UCL injury, *n* (%)	RCL injury, *n* (%)	Dorsal extensor-related abnormality, *n* (%)
Sports	17 (40.5%)	13 (76.5%)	11 (64.7%)	7 (41.2%)
Falls	14 (33.3%)	11 (78.6%)	8 (57.1%)	4 (28.6%)
Motor vehicle accidents	8 (19.0%)	8 (100.0%)	4 (50.0%)	4 (50.0%)
Traction	3 (7.1%)	3 (100.0%)	0 (0.0%)	2 (66.7%)
Total / overall	42 (100.0%)	35 (83.3%)	23 (54.8%)	17 (40.5%)

**Table 3:** Association between injury mechanism and ligament injury severity (collapsed analysis)

Mechanism of injury	UCL low-grade (0–1), *n*	UCL high-grade (≥ 2), *n*	RCL low-grade (0–1), *n*	RCL high-grade (≥ 2), *n*
Sports	10	7	15	2
Falls	5	9	12	2
Motor vehicle accidents	5	3	7	1
Traction	2	1	3	0
Total	22	20	37	5
p-value*	0.49		0.92	

* p-values obtained from Chi-square test for independence analyzing the association between injury mechanism and collapsed injury severity

## Discussion

Collateral ligament injuries of the thumb MCP joint are common, yet the frequency of concomitant RCL and dorsal extensor-related abnormalities in patients referred for ultrasound remains less well defined. In this study of a selected referred cohort, UCL abnormalities were most frequent, identified in 83.3% of patients, followed by RCL abnormalities in 54.8% and dorsal extensor-related abnormalities in 40.5%. Concomitant abnormalities involving more than one stabilizing structure were observed in a substantial proportion of patients. However, associations with reported injury mechanisms were not statistically significant, and the study design does not permit conclusions regarding the true prevalence of these findings in the overall thumb trauma population.

The role of US is reinforced by its availability and ability to evaluate both ligaments and extensor structures. Prior studies confirmed US accuracy for displaced versus non-displaced UCL tears and for distinguishing partial versus complete lesion [8, 12, 15–17]. Routine use of US in acute thumb trauma has already been shown to improve diagnostic sensitivity and avoid unnecessary surgery [16]. The present findings suggest that, when ultrasound is performed for suspected thumb MCP injury, evaluation of the RCL and dorsal extensor-related structures may provide a more complete characterization of the injury pattern. Previous MRI-based investigation of acute UCL injury has demonstrated that concomitant volar-sided abnormalities may occur [3]. The present study could not assess these structures systematically. Although high-frequency ultrasound can depict the volar plate and adjacent sesamoid complex in experienced hands, evaluation at the thumb MCP joint may be technically challenging in routine practice because the volar plate is small and lies in close relation to the sesamoids, where pain, swelling, probe angulation, and acoustic shadowing may reduce confidence. Accordingly, volar structures were not assessed consistently in this retrospective series and were not included in the analysis.

A study by Werner et al. [10] performed a retrospective review of all thumb metacarpophalangeal (MCP) collateral ligament injuries sustained by athletes on a single National Football League (NFL) team over a 23-year period. The authors found that 25% of the athletes sustained a simultaneous combined tear of the UCL and the RCL. They concluded that concurrent UCL and RCL tears represent a distinct and underappreciated injury entity that often necessitates surgical repair. Similarly, Bhat [18] also presented a case of combined RCL and UCL injury in 15 year-old boy with pain and thumb instability who eventually underwent delayed primary repair of both ligaments. In contrast, in the present study cohort, which included varied mechanisms and injury severities, most additional abnormalities were low-grade on ultrasound. Because the ultrasound unit functioned as an imaging service rather than the treating service, it was not possible to determine whether identifying these additional findings altered subsequent management in practice. This supports the concept that collateral and extensor injuries form a continuum of joint trauma, not always requiring operative intervention. The observation that more than one thumb stabilizer was abnormal in a substantial proportion of this selected cohort suggests that some injuries may reflect more complex loading patterns, including a rotational component, rather than pure valgus stress alone. However, this interpretation should be viewed as hypothesis-generating only, since the present study was not designed to define the underlying biomechanics, and isolated UCL and isolated RCL abnormalities were also present.

From a clinical perspective, the role of ultrasound in thumb UCL injury is already well established, particularly for distinguishing displaced from non-displaced tears and for supporting treatment decisions in selected cases [7, 12, 15–17]. Previous MRI-based work has also shown that acute UCL injuries may be accompanied by associated abnormalities around the thumb MCP joint [3]. In this selected referred cohort, most of these additional abnormalities were low-grade, and their main clinical value appears to lie in more complete characterization of the injury pattern, together with improved patient counseling and follow-up.

These results add to prior literature by describing the frequency of concomitant ultrasound abnormalities in a consecutive selected referral cohort. While UCL abnormalities remained the most common finding, more than half of patients also demonstrated RCL or dorsal extensor-related abnormalities.

At present, the principal value of recognizing these low-grade dorsal extensor-related abnormalities is improved diagnostic completeness and counseling. These findings should therefore be interpreted primarily as descriptive imaging findings rather than as independent prognostic markers.

The present study has several important limitations. First, it was a retrospective, single-center series composed only of patients in whom at least one stabilizing structure was abnormal on ultrasound. Excluded cases were related to age younger than 18 years or insufficient data in the medical record. Accordingly, the observed frequencies describe a selected referred cohort and should not be interpreted as prevalence estimates for the overall thumb-trauma population. At the same time, the cohort was consecutive within the predefined eligibility criteria and therefore reflects a real-world referral population encountered in routine practice. Second, all examinations were performed by a single experienced operator, and neither interobserver nor intraobserver reliability could be assessed. However, this also ensured technical consistency across all examinations. Third, standardized clinical examination findings, formal stability testing, hand dominance, and treatment impact were not available in a sufficiently uniform manner for retrospective analysis. In addition, dorsal extensor-related findings were recorded descriptively rather than according to a validated grading system, and because of the close anatomic relationship between the extensor tendon and the dorsal capsule at the thumb MCP joint, retrospective distinction between tendon-related and dorsal capsular-related abnormalities was not always possible. To reduce overinterpretation, these findings were described conservatively as dorsal extensor-related abnormalities. Fourth, the ultrasound unit functioned as an imaging service rather than the treating service, so it was not possible to determine whether the additional abnormalities identified on ultrasound altered subsequent management. Fifth, an independent reference standard, such as MRI, operative findings, or second-examiner ultrasound, was not routinely available, so the diagnostic performance of the additional findings could not be established. Nevertheless, prior studies have shown good diagnostic performance of ultrasound for thumb collateral ligament injury, and most additional abnormalities in the present cohort were low-grade. Finally, volar structures, including the volar plate, were not assessed systematically in this retrospective series and may therefore have been under-recognized.

In conclusion, ultrasound assessment of thumb MCP trauma in this referral cohort showed that injuries were often more complex than an isolated UCL tear. Concomitant RCL and dorsal extensor-related abnormalities were identified in a considerable proportion of patients. When evaluating thumb MCP trauma, systematic assessment of these adjacent stabilizing structures may improve diagnostic completeness and provide a more comprehensive diagnosis.

## Data Availability

No datasets were generated or analysed during the current study.
